# Sparse Representation Based SAR Vehicle Recognition along with Aspect Angle

**DOI:** 10.1155/2014/834140

**Published:** 2014-04-09

**Authors:** Xiangwei Xing, Kefeng Ji, Huanxin Zou, Jixiang Sun

**Affiliations:** College of Electronic Science and Engineering, National University of Defense Technology, Changsha, Hunan 410073, China

## Abstract

As a method of representing the test sample with few training samples from an overcomplete dictionary, sparse representation classification (SRC) has attracted much attention in synthetic aperture radar (SAR) automatic target recognition (ATR) recently. In this paper, we develop a novel SAR vehicle recognition method based on sparse representation classification along with aspect information (SRCA), in which the correlation between the vehicle's aspect angle and the sparse representation vector is exploited. The detailed procedure presented in this paper can be summarized as follows. Initially, the sparse representation vector of a test sample is solved by sparse representation algorithm with a principle component analysis (PCA) feature-based dictionary. Then, the coefficient vector is projected onto a sparser one within a certain range of the vehicle's aspect angle. Finally, the vehicle is classified into a certain category that minimizes the reconstruction error with the novel sparse representation vector. Extensive experiments are conducted on the moving and stationary target acquisition and recognition (MSTAR) dataset and the results demonstrate that the proposed method performs robustly under the variations of depression angle and target configurations, as well as incomplete observation.

## 1. Introduction

In the recent years, sparse representation has attracted much attention in the fields of signal representation, compress sensing, and classification. The sparse representation classification (SRC) algorithm, which is proposed by Wright et al. [1], has boosted the classification method on many subjects such as face recognition [2], hyperspectral image classification [3], and synthetic aperture radar (SAR) automatic target recognition (ATR) [4–6].

In particular, with the development of SAR imaging techniques including high resolution and multipolarization, much effort has been devoted to SAR ATR. The moving and stationary target acquisition and recognition (MSTAR) dataset [7], which collects the SAR images of typical vehicles under the conditions of various radar grazing angles and target aspect angles, is a benchmark for the development and evaluation of recognition algorithms. One of the most popular methods for MSTAR classification is template matching. Ross et al. [8] decomposed the train samples of each class into 36 templates with an aspect range of 10° and then classified the test samples based on the distance measurement compared with the templates. Ravichandran and Casasent [9] proposed the minimum noise and correlation energy (MINACE) filter method to achieve an optimal classification result. The learning vector quantization (LVQ) method [10], which acquires the train samples with learning, is another template matching method. Besides the previous template matching methods that directly applied on image pixels, the feature-based template matching methods improve the performance. Ramamoorthy and Casasent [11] extract the rotation invariant Fourier features and propose a feature space trajectory (FST) classifier. Mishra and Mulgrew [12] investigate the classification of MSTAR targets based on principle component analysis (PCA). Yang et al. [13] summarize and compare the various classifiers for MSTAR target classification.

Through representing the test sample as the combination of training samples, sparse representation classification, which can be considered as a generalization of the LVQ, determines the class of the test sample based on the resulted sparsest coefficients. The sparse coefficients contain discriminatory information of the samples in low-dimensional subspace and are robust to noise and occlusion as well as incomplete observation [2]. However, the target presents diverse appearance and heavy occlusion on SAR image according to the target's aspect angle. The sparse coefficients that lie on a large difference of aspect angle lead to classification errors. In this paper, we propose a novel SAR vehicle classification method based on SRC along with aspect angle (SRCA). The method first estimates the aspect angle of all the samples and solves the sparse representation vector with a dictionary that consists of the PCA features of all the training samples. Then, we project the sparse coefficient vector onto a subspace that is around the test sample's aspect angle. Finally, the method assigns the test sample to a certain category, which minimizes the reconstruction error with the novel sparse representation vector. We validate our proposed method by testing on a subset of the MSTAR dataset. It is shown that the proposed method is superior to the methods of linear SVM, kernel SVM, and the original SRC.

## 2. Sparse Representation Based Classification

In this section, we give a brief review on the sparse representation and the classification strategy, that is, how to represent a test sample as the combination of training samples from a dictionary [14] and determine the class based on the sparse representation vector. Note that the test sample represents a vehicle or other objects such as a face. In this letter, we focus on vehicles.

### 2.1. Sparse Representation [1]

Suppose that there are *K* distinct classes of vehicles and the labeled training samples for the *K* classes are known* a priori*; then, our objective is to correctly determine the class of a new test vehicle sample. Assume that the number of training samples for the *i*th class is *n*_*i*_ and that the dimension of each sample is *m*, and denote *ϕ*_*i*,*j*_ ∈ ℝ^*m*×1^ by the *j*th training sample for class *i*. Then, the matrix for class *i* containing all *n*_*i*_ training samples is given by

(1) $$\begin{array}{c} {{𝚽}_{i}}^{m\times n_{i}}=\left[\phi_{i,1},\phi_{i,2},\ldots ,\phi_{i,n_{i}}\right]. \end{array}$$

Accordingly, all training samples for the *K* classes are concatenated into a dictionary matrix *𝚽* ∈ ℝ^*m*×*N*^; that is,

(2) $$\begin{array}{c} {𝚽}^{m\times N}=\left[{𝚽}_{1},{𝚽}_{2},\ldots ,{𝚽}_{K}\right], \end{array}$$

where *N* = ∑_*i*=1_^*K*^ *n*_*i*_ denotes the total number of training samples. With the dictionary *𝚽* at hand, we consider an observed new test sample denoted by **y** ∈ ℝ^*m*×1^. If this sample belongs to class *i*, then it can be well approximated by a linear combination of the training samples in the *i*th class; that is,

(3) $$\begin{array}{c} y^{m\times 1}=c_{i,1}\phi_{i,1}+c_{i,2}\phi_{i,2}+\cdots +c_{i,n_{i}}\phi_{i,n_{i}} \end{array}$$

in which the scalar *c*_*i*,*j*_ is the weighted coefficient associated with the *j*th training sample of class *i* to reconstruct the sample **y**. Correspondingly, the linear representation of **y** using all training samples in the dictionary can be denoted by

(4) $$\begin{array}{c} {\left[y\right]}^{m\times 1}={\left[𝚽\right]}^{m\times N}{\left[x\right]}^{N\times 1} \end{array}$$

with **x** = [0,…,0,*c*_*i*,1_,…,*c*_*i*,*n*_*i*__,0,…,0]^*T*^ being a sparse weighted coefficient vector whose entries are all zero except those associated with the *i*th class.

With a sufficiently large number of samples for each class, the coefficient vector **x** is expected to be very sparse. Based on the recent development in the theory of sparse representation and compressive sensing, the solution of **x** can be recovered via solving the following *ℓ* ^1^-norm minimization problem [1, 2]:

(5) $$\begin{array}{c} \underset{x}{\min}{||x||}_{1}\;\text{subject}\;\text{to}\;y=𝚽x, \end{array}$$

where ||**x**||_1_ denotes the *ℓ* ^1^-norm of **x**, which sums up the absolute values of all entries in **x**. Moreover, the equality constraint in (5) can be relaxed to allow noise; that is, problem (5) can be relaxed as

(6) $$\begin{array}{c} \underset{x}{\min}{||x||}_{1}\;\text{subject}\;\text{to}\;||y-𝚽x||\leq \varepsilon , \end{array}$$

where *ε* is the allowed error tolerance. Problems (5) and (6) can be recast as linear programs (LP) and second-order cone programs (SOCP), respectively. Thus, they are both convex and can be solved by existing convex optimization software [15]. It is worth noting that the complexity of solving the SOCP is *O*(*m* ^2^ *N* ^2^), where *m* and *N* are the dimension of the sample and the total number of training samples, respectively (c.f. (1), (2)).

### 2.2. Sparse Representation Based Classification

With the sparsest coefficient ${\hat{x}}_{1}$ at hand, the SRC method determines the class of the test sample **y** in the following. Ideally, if all the nonzero entries in the estimate ${\hat{x}}_{1}$ are associated with one single class *i*, then we can easily determine the *i*th class that the test sample belongs to. However, due to modeling error and noise, small nonzero entries associated with multiple other classes may exist. To tackle this challenge, the classification strategy in [1] is as follows to harness the subspace structure of  ${\hat{x}}_{1}$ for classification.

For each class *i*, let *δ*_*i*_ : ℝ^*n*^ → ℝ^*n*^ be the characteristic function that selects the coefficients associated with the *i*th class. That is, for any vector  **x** ∈ ℝ^*n*^, *δ*_*i*_(**x**) ∈ ℝ^*n*^ is a new vector whose nonzero entries are only the entries in **x** associated with class *i*. Then, we can reconstruct the test sample as ${\hat{y}}_{i}=𝚽\delta_{i}\left({\hat{x}}_{1}\right)$ and recognize **y** as the class that has the minimum residual between **y** and ${\hat{y}}_{i}$; that is,

(7) $$\begin{array}{c} \arg\underset{i}{\min}\;r\left(y\right)={||y-𝚽\delta_{i}\left({\hat{x}}_{1}\right)||}_{2}. \end{array}$$

**Figure 1:**
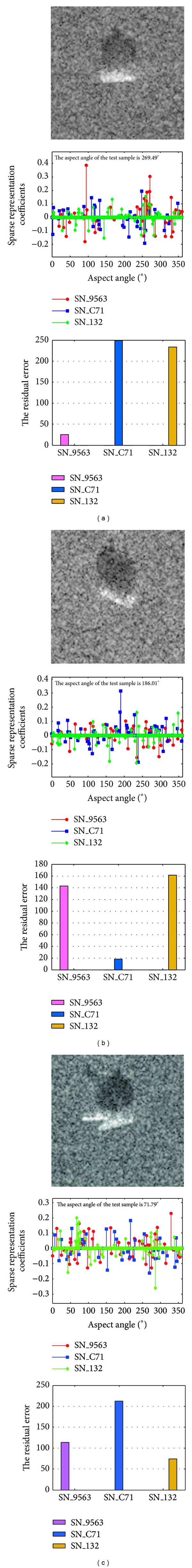
Sparse representation examples for 3 different test targets: (a) SN_9563, (b) SN_C71, and (c) SN_132. From top to bottom: input test target, the corresponding sparse representation coefficient vector, and the reconstruction residual error. All the targets are represented by colors and markers distinct for each class, with red circle for SN_9563, blue square for SN_C71, and green diamond for SN_132, as shown in legend.

In Figure 1, we present an example for sparse representation based classification method. In this illustration, we use the first 3 classes (SN_9563 from BMP2, SN_C71 from BTR70, and SN_132 from T72) from the MSTAR database. Detailed description of the MSTAR database is given in Section 4. The dictionary used in the experiment consists of the training data from the 3 classes. The sparse representation coefficient vector for 3 test images from each class is shown in Figure 1, recovered by solving (6) using the algorithm described in [15]. As shown in Figure 1, for each test image, the recovered sparse coefficient vector has most of its nonzero elements concentrated at the ground-truth class, and the resulting residual error for the same class is minimum. Therefore, the class of the test image is determined by the sparse representation coefficient vector.

## 3. SRC along with Aspect Angle

In SAR images, even the same target presents different appearances with the variation of aspect angle. In this section, the aspect information is evaluated for the classification of vehicles in SAR image. Based on the analysis of the correlation of the test image with the train images of various aspects, the sparse representation vector is mapped onto a local aspect range and the algorithm of SRC along with aspect angle is proposed.

### 3.1. Correlation Analysis

The correlation between two images reflects the similarity of them. A higher correlation coefficient means the two target images are likely to come from the same class. Based on the correlation coefficient, the template matching method has been widely adopted in SAR ATR [16]. By calculating the correlation of the test image with the training images of various aspects, we here evaluate the essentiality of introducing the aspect information for the vehicle classification in SAR images.

Given two images, the correlation coefficient is calculated as follows [17]:

(8) $$\begin{array}{c} R_{A,B}=\underset{m,n}{\max}\frac{\sum_{x}\sum_{y}\left[A\left(x,y\right)-\overset{-}{A}\right]\left[B\left(x-m,y-n\right)-\overset{-}{B}\right]}{{\left[\sum_{x}\sum_{y}{\left[A(x,y)-\overset{-}{A}\right]}^{2}{\left[B(x,y)-\overset{-}{B}\right]}^{2}\right]}^{1/2}} \end{array}$$

in which **A** is the test image, **B** is the train image, *m* and *n* are the offsets on the direction of range and azimuth separately, and the shift aligns the target area in the image. The numerator in (8) is a convolution procedure, which can be achieved efficiently by multiplications in Fourier domain.

**Figure 2:**
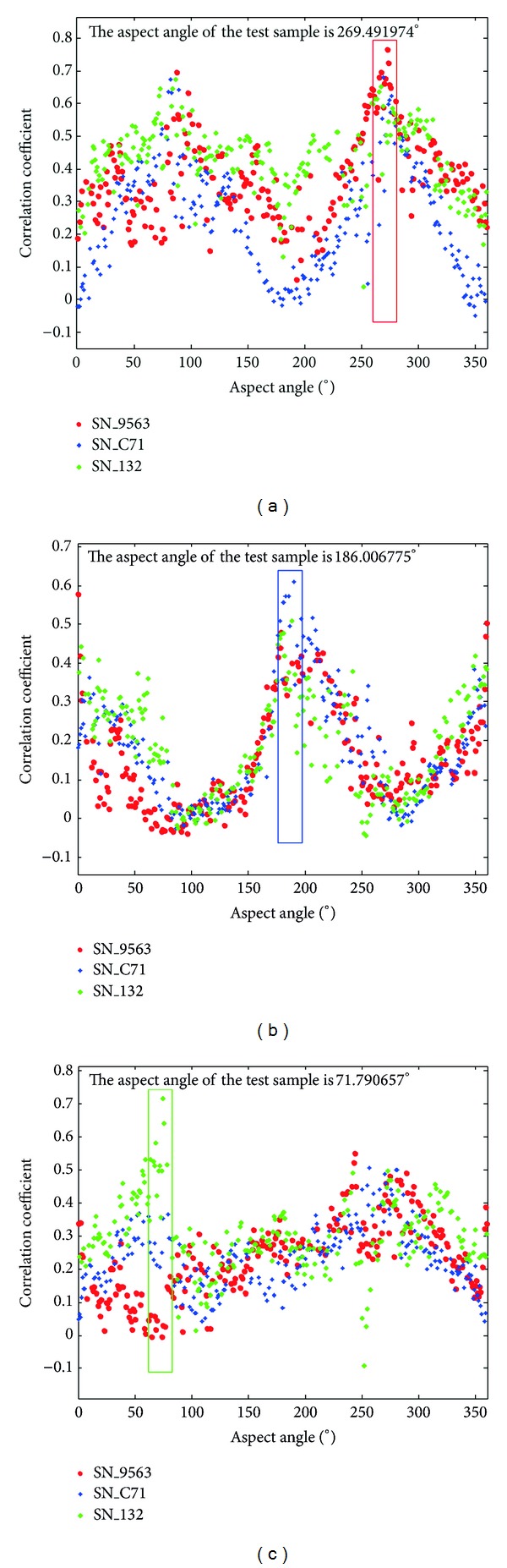
Correlation coefficients of the input targets in Figure 1 with different class of train samples. (a) SN_9563, (b) SN_C71, and (c) SN_132. The rectangle indicates a local aspect range of 17° around the aspect of the test sample.

Figure 2 illustrates the correlation coefficients of 3 different test images with the train images from distinct classes. In general, the correlation coefficients of the test image with the same class of train samples are larger than with the other two classes. In particular, the test image presents high correlation with the train samples within a local aspect range, as indicated by the rectangle in Figure 2. Therefore, the aspect information is expected to be utilized in the SAR ATR.

### 3.2. Mapping the Sparse Representation Vector onto a Local Aspect Range

**Figure 3:**
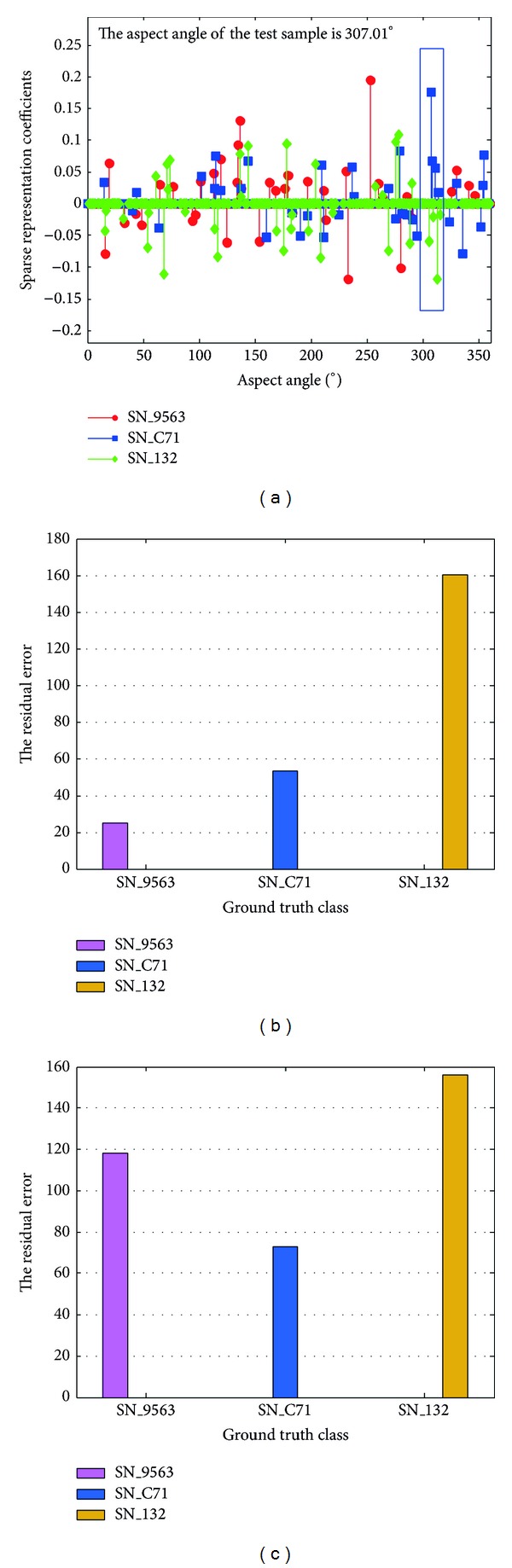
The sparse representation vector and residual error of a test sample from SN_C71. (a) The sparse representation vector. (b) Residual error calculated with the complete sparse representation vector. (c) Residual error calculated with the sparse coefficients within a certain aspect range. The ground-truth aspect angle of the test sample is 307.01°, and the rectangle indicates the neighboring range around the aspect of the test sample.

The vehicles in SAR images are aspect sensitive and the test sample is more likely represented by the train sample whose aspect angle is close to the test sample's. The conclusion is preliminarily validated by the correlation coefficients in Figure 2. Moreover, we present a sparse representation vector that leads to incorrect result of classification in Figure 3. The ground truth class of the test sample is SN_C71. When the sparse coefficients on the whole aspect space are adopted, the reconstruction residual error is the least for the class of SN_9563 instead of SN_C71. If we concentrate the sparse coefficient vector on a local range of aspect that is around the aspect of the test sample, the resulting residual error of the class of SN_C71 is the minimum and an improved classification result is observed.

Motivated by the above observations and analysis, we propose the SRC method along with aspect angle. For each class *i*, we redefine a characteristic function *δ*_*i*,*ψ*_*y*_,*ψ*_*r*__ : ℝ^*n*^ → ℝ^*n*^ that selects the coefficients associated with the *i*th class and a certain range *ψ*_*r*_ of the test sample's aspect angle *ψ*_*y*_. Then, similar with the original SRC, the class of the test sample **y** is determined with the minimum residual:

(9) $$\begin{array}{c} \arg\underset{i}{\min}\;r\left(y\right)={||y-𝚽\delta_{i,\psi_{y},\psi_{r}}\left({\hat{x}}_{1}\right)||}_{2}. \end{array}$$

It should be noticed that the aspect information can also be introduced to the SRC by other alternative ways, such as constructing the dictionary with the train samples of certain aspect or taking the aspect angle as one of the rows of the dictionary. However, the first one requires a large number of train samples of certain aspect to construct the overcomplete dictionary, and the second one is limited by the different dimensions of the aspect and other atoms in the dictionary. Therefore, we intuitively map the sparse coefficient vector onto a local range of aspect and calculate the residual error with the tailored sparse vector. The effectiveness of the proposed method will be further validated in Section 4.

### 3.3. Sparse Representation Based Classification with Aspect Angle

**Figure 4:**
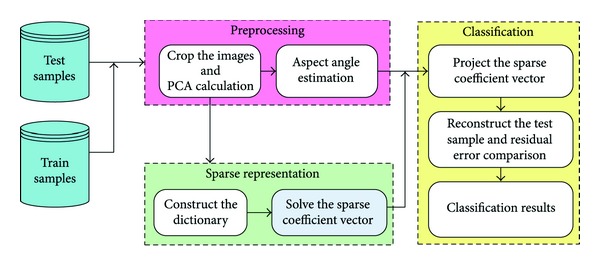
Procedure diagram of the proposed SRCA method.

The proposed SAR vehicle classification method consists of three modules: (1) preprocessing, including cropping the image size, principle component analysis (PCA) feature extraction, and estimating the aspect angle; (2) sparse representation of the test sample with a constructed dictionary; and (3) determining the class of the test sample based on the sparse representation vector and aspect angle. The overall procedure of the proposed sparse representation classification along with aspect angle (SRCA) method is summarized in Figure 4.

In the first module, the aspect angle of the vehicle in SAR image is estimated through image processing techniques. Firstly, the target area is separated from the background with segmentation methods [18]. Then, the minimized enclose rectangle (MER) is calculated and therefore the aspect angle is estimated. In this procedure, there exists an uncertainty of 180° of the estimated aspect angle. We eliminate the ambiguity by assuming the tailstock section to be the border section with the highest mean RCS value. In the following two processing modules, the sparse representation vector is achieved by resolving (6), and the reconstruction residual error is calculated with (9).

## 4. Experiment Results

In this section, we evaluate the performance of the proposed method using MSTAR public database, which is a standard dataset for evaluating SAR ATR algorithms, and collected in 1995 and 1996 by the Sandia National Laboratory X-band (9.6 GHz) HH-polarization SAR sensor with the resolution of 0.3 m × 0.3 m. One subset of the MSTAR data consists of three classes of vehicles, that is, the BMP2, BTR70, and T72, with several configuration variations for each class. The vehicles are imaged in spotlight mode at 15° and 17° depression angles over 360° of aspect angles. The capacity of the subset is illustrated in Table 1. Since the original image dimension is very high (128 × 128 = 16384), we crop the data to 64 × 64 and use the PCA method [12, 19] for feature extraction to reduce their dimensionality. Other feature extraction methods such as downsampling, Gaussian random projection [1], and Manifold learning [20] are also applicable to the SRC. For comparison, we compare with several state-of-the-art classification methods: linear SVM, kernel SVM (KSVM) with radial basis function (RBF) kernel, and SRC. For both linear SVM and KSVM, the LIBSVM package [21] is adopted. The radius for the RBF of KSVM is empirically set as *σ* = 4, and the tolerance error in (6) is set as *ε* = 0.05.

In the sequel, we carry out several experiments. Firstly, we evaluate the performance of the proposed method under different adopted range of aspect and feature dimensionalities. We then examine the robustness of the proposed method with respect to the variations of depression angle and target configurations. Finally, we evaluate the proposed algorithm under the condition of incomplete observation.

### 4.1. Performance on Different Adopted Range of Aspect and Dimensionalities

In this experiment, we use the first serial number targets from each class, that is, SN_9563 for BMP2, SN_C71 for BTR70, and SN_132 for T72, for algorithm evaluation and comparison. The training samples are captured at depression angle of 17° and the testing samples are captured at depression angle of 15°.

**Figure 5:**
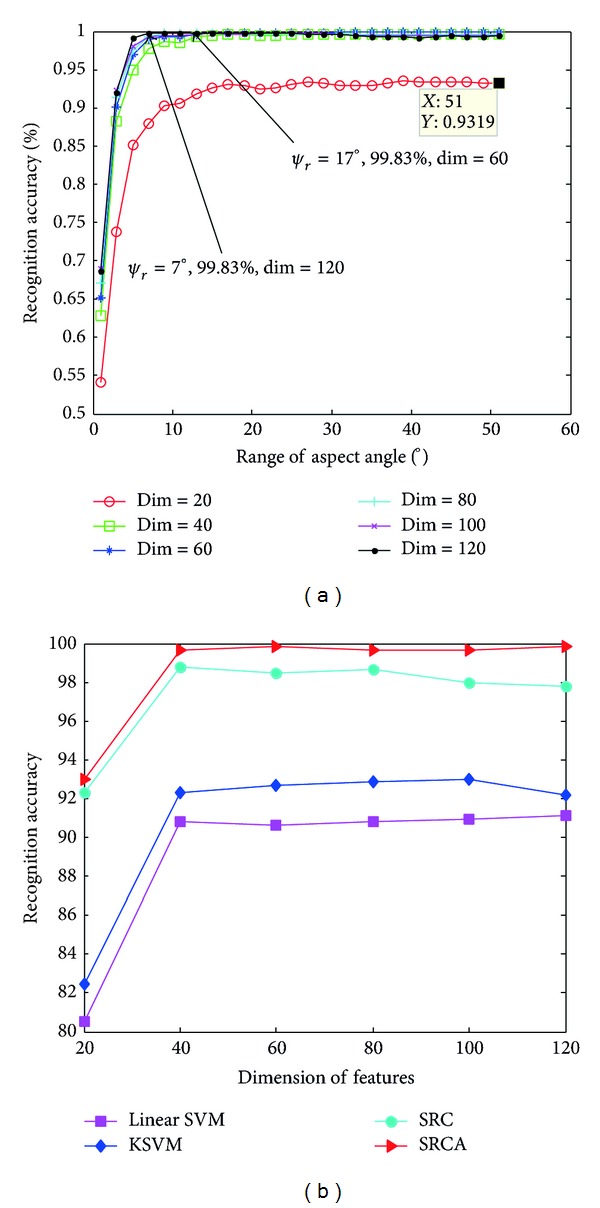
Recognition performance of different algorithms under (a) different adopted range of aspect angle with *d* = 144 and (b) different feature dimensions with *ψ*_*r*_ = 17°.

In our first experiment, we evaluate the recognition accuracy of the proposed SRCA method via different range of aspect for the different feature dimensions. The performance curves in Figure 5(a) illustrate that the recognition accuracy of the SRCA method varies with the adopted aspect range. At the beginning, the accuracy increases as fast as the adopted aspect range increases and retains a high level for several aspect range. However, when the aspect range keeps on increasing, the performance presents some degradation, which validates the effectiveness of carrying the classification on a certain range of aspect. When the feature dimension is *d* = 60, the proposed method achieves best performance under the condition that the aspect range is *ψ*_*r*_ = 17°.

In the following experiment, we compare the performance of different algorithms when feature dimension changes. The corresponding results are summarized in Table 2 and a graphical plot is given in Figure 5(b) for visualization. As can be seen from Table 2 and Figure 5(b), the SRCA and SRC methods outperform the SVM methods by a notable margin. As the feature dimension increases, the proposed SRCA method achieves saturation faster than the other methods. Even when the performance of SRC represents little degradation, the performance of SRCA is still desirable. This once again verifies the effectiveness of introducing the aspect information for SAR ATR.

### 4.2. Depression Angle Invariance

For the real-world tasks, the invariance to depression angle is crucial to the successful application of a recognition algorithm. In this subsection, we evaluate the invariance to depression angle for the four algorithms. There are two different depression angles for the first 3 classes of MSTAR, that is, 17° and 15°. In the previous experiment, we have taken the samples captured on the depression angle of 17° for training and the samples captured on the depression angle of 15° for testing. In this experiment, we exchange the testing and training samples. As can be seen from Table 3, all the methods perform some degradation when the samples of 15° depression angle are used for training, which illustrates that the depression angle is important for the recognition task. However, the proposed SRCA method is still superior to the other methods.

### 4.3. Configuration Invariance

In this subsection, we examine the invariance of different algorithms under different configurations, which is a desirable property of an algorithm for SAR ATR applications. As shown in Table 1, the BMP2 and T72 both have different configurations of images captured from different variants of the same vehicle type. We compare the results of different algorithms according to the following settings: for training, the images from SN_9563 for BMP2, SN_C71 for BTR70, and SN_132 for T72 at the depression angle of 17° are used. For testing, the configuration of SN_C71 for BTR70 is used and 3 different testing sets for the other classes are used at the depression angle of 15°: (1) invariant: SN_9563 for BMP2 and SN_132 for T72; (2) mixed: all the images of BMP2 and T72 from all the 3 variants; (3) variant: carrying out a test on SN_9566 and SN_C21 for BMP2 and SN_812 and SN_S7 for T72.The classification results are summarized in Table 4. For the invariant case, the proposed SRCA method achieves a highest classification rate of 99.83%, which is much better than all the other methods. When testing the dataset with different configurations (“variant”), the proposed method can still achieve a recognition rate of 87.37%. In particular, for the configuration variants of BMP2, the degradation is acceptable and is better than the other methods. The results in this subsection further validate the effectiveness of the proposed method.

### 4.4. Incomplete Observation Invariance

**Figure 6:**
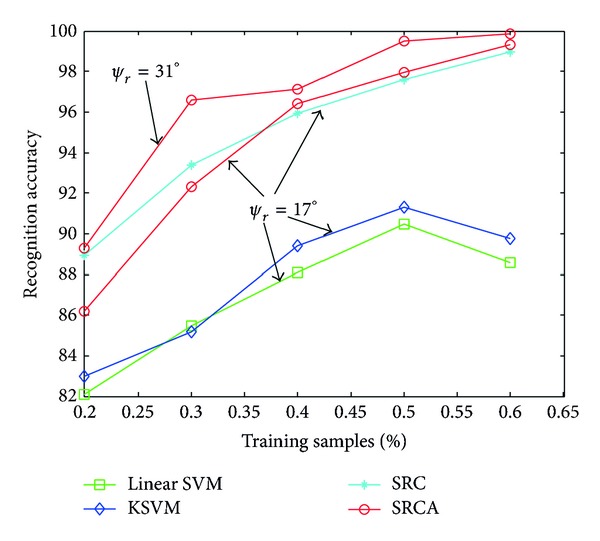
Performance comparison of the algorithms under incomplete observation.

In the real-world tasks, the targets are not observed under all conditions, such as every aspect angles, radar frequencies, and grazing angles. The incomplete observation proposes challenges to the recognition algorithms. We evaluate the robustness of proposed SRCA method under the condition of incomplete observation. In this experiment, the training samples captured at the depression angle of 17° are selected randomly with a certain percentage to construct the training set, and the samples captured at the depression angle of 15° are tested. The performances of different methods are compared in Figure 6. The SRC based methods perform better than the SVM based methods by a notable margin. When the percentage is small, the absence of majority of training samples degrades the performance of SRCA. As the percentage increases, the performance of SRCA outperforms the SRC method and performs the best among the four methods. Another worthwhile point to note is that the performance of SRCA method improves with the increase of the adopted range of aspect angle, as shown in Figure 6 (*ψ*_*r*_ = 31° compared to *ψ*_*r*_ = 17°).

## 5. Conclusions

In this paper, we propose a SAR vehicle recognition method based on sparse representation classification along with aspect angle. The method projects the sparse coefficient vector onto a subspace that is within a certain range of aspect angle around the estimated aspect angle of the test sample and then determines the class label according to the reconstruction residuals. The rationality of the idea lies in that the vehicles on SAR image are sensitive to its aspect angle and they are much more likely represented by the training samples with similar aspect angles. The proposed SRCA method is compared with the linear SVM, KSVM, and SRC methods by carrying extensive experiments on the MSTAR database. The results validate that the proposed SRCA method is robust to the variation of depression angles and target configurations, as well as the incomplete observation of training samples. Despite the effectiveness of the proposed method, much development needs to be further considered in the future work, including the learning of a more compact dictionary from the training data and the fast and effective solution of the sparse representation vector.

## Figures and Tables

**Table 1:** Capacity of the subset of MSTAR.

	BMP2			BTR70	T72		
	SN_9563	SN_9566	SN_C21	SN_C71	SN_132	SN_812	SN_S7
17° (train)^1^	233	[232]	[233]	233	232	[231]	[228]
15° (test)	195	196	196	196	196	195	191

Note: ^1^the samples corresponding to the numbers in brackets are not used in training or testing, unless notified.

**Table 2:** Recognition accuracy (%) on MSTAR with different feature dimensions (*ϕ*_train_ = 17°, *ϕ*_test_ = 15°, and *ψ*_*r*_ = 17°).

Dims. (*d*)	20	40	60	80	100	120	Avg.
Linear SVM	80.56	90.79	90.62	90.79	90.96	91.14	89.14
KSVM	82.43	92.33	92.67	92.84	93.01	92.16	90.91
SRC	92.33	98.81	98.47	98.64	97.96	97.78	97.33

SRCA	93.01	99.66	99.83	99.66	99.66	99.83	**98.61**

**Table 3:** Depression angle invariance results (%) for different algorithms (*d* = 60 and *ψ*_*r*_ = 17°).

Datasets	Linear SVM	KSVM	SRC	SRCA
*ϕ*_train_ = 17°	90.62	92.67	98.47	**99.83**
*ϕ*_train_ = 15°	89.40	91.98	97.85	**99.14**

**Table 4:** Configuration invariance results (%) for different algorithms (*ϕ*_train_ = 17°, *ϕ*_test_ = 15°, *d* = 60, and *ψ*_*r*_ = 17°).

Algorithms	Datasets	Invariant			Mixed			Variant		
	Input ↓	BMP2	BTR70	T72	BMP2	BTR70	T72	BMP2	BTR70	T72
Linear SVM	BMP2	85.13	8.21	6.66	75.13	10.05	14.82	71.17	10.20	18.62
	BTR70	2.55	92.43	1.02	2.55	94.90	2.55	1.53	96.94	1.53
	T72	7.65	2.04	90.31	16.49	10.31	73.20	20.98	11.66	67.36
	Avg.	90.62			77.14			74.85		

KSVM	BMP2	88.21	7.18	4.61	76.32	10.73	12.95	74.74	9.95	15.30
	BTR70	2.55	96.94	0.51	2.04	95.92	2.04	1.53	97.45	1.02
	T72	6.12	1.02	92.86	14.60	5.84	79.55	19.95	5.18	74.87
	Avg.	92.67			80.51			79.36		

SRC	BMP2	97.44	0	2.56	90.97	2.04	6.98	86.48	3.83	9.69
	BTR70	1.53	97.96	0.51	0.51	98.98	0.51	0	100	0
	T72	0	0	100	6.87	4.12	**89.00**	10.36	4.15	**85.49**
	Avg.	98.47			**92.98**			**88.81**		

SRCA	BMP2	**100**	0	0	**93.70**	2.38	3.92	**91.84**	4.34	3.83
	BTR70	0.51	**99.49**	0	0	**100**	0	0	**100**	0
	T72	0	0	**100**	10.14	5.84	84.02	15.54	8.03	76.42
	Avg.	**99.83**			90.48			87.37		
